# Soluble CD40 Levels in Rheumatoid Arthritis: Association with Serum Cytokines and Autoantibody Status

**DOI:** 10.3390/jcm15155836

**Published:** 2026-07-26

**Authors:** Valeria Miroslava Limón-López, José Francisco Muñoz-Valle, Zyanya Reyes-Castillo, Gloria Esther Martínez-Bonilla, Edith Oregon-Romero, Samuel García-Arellano, Christian Johana Baños-Hernández, Ilce Valeria Román-Fernández

**Affiliations:** 1 Doctorado en Ciencias Biomédicas (DCB), Centro Universitario de Ciencias de la Salud, Universidad de Guadalajara, Guadalajara 44340, Jalisco, Mexico; miroslava.limon@alumnos.udg.mx; 2 Instituto de Investigación en Ciencias Biomédicas (IICB), Centro Universitario de Ciencias de la Salud, Universidad de Guadalajara, Guadalajara 44340, Jalisco, Mexico; biologiamolecular@hotmail.com (J.F.M.-V.); edith.oregon@academicos.udg.mx (E.O.-R.); samuel.garcia4566@academicos.udg.mx (S.G.-A.); johana.banos@academicos.udg.mx (C.J.B.-H.); 3 Instituto de Investigaciones en Comportamiento Alimentario y Nutrición (IICAN), Universidad de Guadalajara, Ciudad Guzmán 49010, Jalisco, Mexico; zyanya.reyes@cusur.udg.mx; 4 Servicio de Reumatología, Organismo Público Descentralizado Hospital Civil de Guadalajara “Fray Antonio Alcalde”, Guadalajara 44280, Jalisco, Mexico; glomarbon@hotmail.com

**Keywords:** rheumatoid arthritis, sCD40, autoantibodies, cytokines, disease progression

## Abstract

**Background**: In rheumatoid arthritis (RA), the CD40-CD40L axis plays a key role in immune cell activation and the production of inflammatory mediators. Although the soluble form of CD40 (sCD40) has been identified and investigated in various autoimmune disorders, its significance in RA and its relationship with inflammatory and humoral markers remain to be fully elucidated. **Methods**: Sixty-two patients with RA, classified according to the 2010 ACR/EULAR criteria, and 31 age- and sex-matched healthy controls were included. Clinical characteristics, disease activity (DAS28-ESR), and acute-phase reactant levels (CRP and ESR) were evaluated in patients with RA. Autoantibodies (RF, ACPA, anti-MCV, and anti-PAD4), serum cytokines, and sCD40 levels were quantified using ELISA and multiplex bead-based assays. Group differences and associations were assessed using nonparametric tests. Multiple linear regression analysis was performed to account for potential confounding variables. **Results**: Serum sCD40 levels did not differ significantly between patients with RA and healthy controls or among patient subgroups stratified by disease activity or sex. No associations were observed between sCD40 levels and autoantibody seropositivity or titers. sCD40 levels were positively correlated with age (*r_s_* = 0.293, *p* = 0.02). Notably, after adjustment for age, sCD40 showed a significant negative correlation with IL-2 (*r_s_* = −0.317, *p* = 0.01). **Conclusions**: Serum sCD40 does not reflect disease activity or the humoral immune response in RA. Nevertheless, further studies are needed to evaluate its relevance during early stages of the disease and to further characterize its role in cytokine production.

## 1. Introduction

Rheumatoid Arthritis (RA) is a chronic inflammatory autoimmune disease characterized by synovial hyperplasia, bone erosion, and cartilage destruction in peripheral joints, ultimately leading to functional impairment. The disease is characterized by the presence of autoantibodies, primarily rheumatoid factor (RF) and anti-citrullinated peptide antibodies (ACPA), together with elevated concentrations of proinflammatory cytokines such as IL-6, TNF-α, IL-17, and IL-1β [1,2]. The production of inflammatory effector molecules in RA is largely dependent on costimulatory signaling, with the CD40-CD40L pathway playing a central role [3,4,5].

CD40 (UniProt ID P25942) is a 48 kDa transmembrane receptor belonging to the tumor necrosis factor receptor superfamily (TNFRSF) that is predominantly expressed on antigen-presenting cells, including B cells, dendritic cells, and macrophages. However, CD40 expression has also been identified in various non-immune cells, including platelets, endothelial cells, fibroblasts, and epithelial cells [3,4,5]. CD40L (CD154, UniProt ID P29965), in contrast, is a 32–39 kDa member of the tumor necrosis factor superfamily (TNFSF). It is primarily expressed by activated T cells but is also found on activated B cells and platelets. Under inflammatory conditions, CD40L expression can be induced in monocytes, natural killer cells, basophils, and mast cells [3,4,5].

During adaptive immune responses, CD40-CD40L interactions are essential for multiple B-cell functions, including germinal center formation, immunoglobulin class-switch maturation, memory B-cell generation, and regulation of apoptosis [3,4,5]. Furthermore, this pathway promotes the production of proinflammatory mediators, with the specific response depending on the cell type in which CD40 signaling is activated. These mediators include TNF-α, IL-8, MIP-1α, MCP-1, IL-6, IL-12, IL-17, and IL-1β [3,4,5].

In addition to their membrane-bound forms, soluble forms of both CD40 (sCD40) and CD40L (sCD40L) have been characterized. The release of sCD40 and sCD40L is known to occur through enzymatic proteolytic cleavage [6,7,8,9,10]; in the case of sCD40, alternative splicing has also been described [11,12]. Owing to its cytokine-like properties, elevated circulating sCD40L levels have been reported in several conditions, including COVID-19 [13], acute coronary syndrome [14], type 1 diabetes mellitus (T1DM) [15], and systemic lupus erythematosus (SLE) [16]. Likewise, increased sCD40 levels have been described in several autoimmune and inflammatory diseases, including Graves’ disease (GD) and Hashimoto’s thyroiditis (HT), in which they correlate with autoantibody markers [17]; psoriasis, regardless of treatment or disease severity [18]; systemic sclerosis (SSc), particularly the limited cutaneous subtype [19]; and chronic urticaria, specifically in autologous serum skin test (ASST)-positive patients [20]. Collectively, these findings highlight the relevance of the soluble CD40-CD40L axis across immune-mediated diseases.

The CD40-CD40L axis has also been reported to be dysregulated in RA, particularly within the synovial microenvironment. Increased CD40 expression has been reported in macrophages [21], fibroblasts [22], monocytes [23], and chondrocytes [24] derived from the joints of patients with RA. In addition, CD40-CD40L interactions in synovial fibroblasts promote cell proliferation and *receptor activator of nuclear factor κB ligand* (*RANKL*) expression, thereby enhancing osteoclastogenesis [25]. CD40-CD40L signaling also directly modulates adaptive immune responses, particularly through B lymphocytes. In this regard, CD40 activation induces B-cell proliferation, differentiation, and immunoglobulin production [26], while also promoting the secretion of proinflammatory cytokines such as IL-8, IL-6, and TNF-α [27].

To date, research in RA has focused primarily on the membrane-bound forms of CD40 and CD40L. However, their soluble counterparts also retain biological activity. sCD40 has been shown to act as a competitive antagonist that inhibits CD40-mediated B-cell responses [28]. In contrast, sCD40L functions as an agonist capable of inducing B-cell proliferation and differentiation comparable to those elicited by its membrane-bound counterpart [29]. Given the established role of the membrane-bound CD40-CD40L axis in RA and the demonstrated biological activity of its soluble forms, characterizing sCD40 and sCD40L and their associations with the clinical features of RA is of considerable interest. In this context, Gutierrez et al. (2019) reported lower serum sCD40 levels in an independent cohort of patients with RA than in healthy controls [30]. Similarly, our group previously evaluated CD40 and CD40L expression in both their membrane-bound and soluble forms in a cohort of patients with RA and examined their associations with clinical characteristics and DAS28 scores [31]. However, neither study evaluated the relationship between sCD40 and proinflammatory cytokines or autoantibody seropositivity, and both included relatively small patient cohorts. Therefore, further studies involving larger cohorts are needed to better characterize these associations and clarify the role of sCD40 in RA.

This study aimed to characterize serum sCD40 levels in patients with RA and evaluate their associations with proinflammatory cytokines and autoantibody seropositivity, thereby contributing to a better understanding of the CD40-CD40L axis in this disease.

## 2. Materials and Methods

### 2.1. Subjects

The study included 62 patients with RA classified according to the 2010 ACR/EULAR criteria and 31 age- and sex-matched healthy controls (HCs) with no history of autoimmune or rheumatic disease and no recent infections. Patients with RA were recruited from the Rheumatology Service of O.P.D. Hospital Civil de Guadalajara “Fray Antonio Alcalde”, Guadalajara, Jalisco, Mexico. Among the patients with RA, a subgroup of 15 untreated patients (off-treatment, OT) was included. These patients were defined as individuals who had not received disease-modifying antirheumatic drugs (DMARDs) or corticosteroids during the preceding 6 months. Patients with other inflammatory, infectious, or rheumatic diseases were excluded.

All procedures were conducted in accordance with the ethical principles of the Declaration of Helsinki. Written informed consent was obtained from all participants before enrollment. The study was approved by the institutional ethics committees of the Centro Universitario de Ciencias de la Salud, Universidad de Guadalajara (approval No. 0122017).

### 2.2. Clinical Assessment

All patients with RA underwent a clinical evaluation performed by a rheumatologist. Clinical and demographic data were collected at the time of enrollment. Disease activity was assessed using the 28-joint Disease Activity Score based on erythrocyte sedimentation rate (DAS28-ESR). Peripheral blood samples were collected from both groups to determine erythrocyte sedimentation rate (ESR) using the Wintrobe method and C-reactive protein (CRP) levels using a high-sensitivity turbidimetric assay (BioSystems, Barcelona, Spain).

### 2.3. Autoantibodies Quantification

To evaluate the association between sCD40 levels and markers of the humoral immune response, serum titers of RF, anti-citrullinated peptide antibodies (ACPA), anti-peptidyl arginine deiminase 4 (anti-PAD4) antibodies, and anti-mutated citrullinated vimentin (anti-MCV) antibodies were quantified in patients with RA.

Serum samples were collected from peripheral blood and stored at −20 °C until analysis. RF (IgM, IgG, and IgA) was measured using a turbidimetric assay (BioSystems, Barcelona, Spain), and quantification was performed on an automated analyzer (BS-120, Mindray, Shenzhen, China). The remaining autoantibodies were measured by ELISA as follows: ACPA (IgG), second-generation ELISA (Axis-Shield Diagnostics, Dundee, UK); anti-MCV antibodies (IgG) (Orgentec Diagnostika, Mainz, Germany); and anti-PAD4 antibodies (IgM, IgG, and IgA) (Cayman Chemical, Ann Arbor, MI, USA). The cut-off values for anti-MCV and anti-PAD4 antibodies were 20 U/mL and 4749 U/mL, respectively, as previously described [32].

### 2.4. Cytokine Quantification

Serum levels of IL-1β, IL-10, IL-13, IL-6, IL-12, IL-17, IFN-γ, TNF-α, IL-2, and IL-4 were quantified using a bead-based multiplex assay (Cytokine Human Magnetic Custom 10-Plex Panel; Invitrogen Life Technologies, Frederick, MD, USA). Serum samples were stored at −80 °C until analysis. Samples were processed according to the manufacturer’s instructions, and fluorescence was acquired using a MAGPIX^®^ Luminex system (Luminex Corp, Austin, TX, USA).

### 2.5. Soluble CD40 Quantification

Serum samples were collected from peripheral blood and stored at −20 °C until analysis. Serum sCD40 levels were quantified using a commercially available ELISA kit (R&D Systems, Minneapolis, MN, USA). The assay had a detection range of 19.5–1250 pg/mL and a sensitivity of 0.54 pg/mL. All samples were analyzed in duplicate using a Multiskan^TM^ Go microplate reader (Thermo Fisher Scientific, Waltham, MA, USA).

### 2.6. Statistical Analysis

Statistical analyses were performed using GraphPad Prism version 8.0, IBM SPSS Statistics version 26.0, and Microsoft Excel 2011. Data distribution was assessed using the Shapiro–Wilk test. Normally distributed continuous variables are presented as the mean ± standard deviation (SD), whereas non-normally distributed variables are presented as the median and interquartile range (IQR; 25th–75th percentile). Categorical variables are presented as absolute frequencies and percentages.

Comparisons between two groups were performed using the Mann–Whitney U test, whereas comparisons among three or more groups were performed using the Kruskal–Wallis test. Correlations were assessed using Spearman’s rank correlation coefficient.

Multiple linear regression analyses were performed to evaluate the association between serum cytokine levels and sCD40 concentrations in patients with RA while adjusting for age as a potential confounding variable. Separate regression models were fitted for each cytokine of interest. Unstandardized regression coefficients (B), 95% confidence intervals (CI), and *p*-values are reported for each model. A two-sided *p*-value < 0.05 was considered statistically significant.

## 3. Results

### 3.1. Clinical Characteristics of Patients with RA

Demographic and clinical characteristics of patients with RA are summarized in Table 1. A total of 62 patients with RA were enrolled, with a mean age of 46.4 ± 10.5 years. The mean age of the healthy control (HC) group was comparable (42.65 ± 9.97, *p* > 0.05). Among patients with RA, the mean disease duration was 4.29 ± 5.98 years. CRP and ESR levels were significantly higher in patients with RA than in healthy controls (61.42 ± 83.34 vs. 2.33 ± 3.55 mg/L and 33.97 ± 18.52 vs. 21.14 ± 11.24 mm/h, respectively; *p* < 0.05). According to DAS28-ESR scores, the patients had moderate disease activity on average (4.2 ± 1.4). Most patients were seropositive for RF, ACPA, and anti-MCV antibodies (69.35%, 62.90% and 54.84%, respectively), whereas most were seronegative for anti-PAD4 antibodies (80.65%). At enrollment, 47 patients were receiving DMARDs in combination with nonsteroidal anti-inflammatory drugs (NSAIDs) or low-dose corticosteroids. The demographic and clinical characteristics of the off-treatment (OT) patients with RA and healthy controls are presented in Supplementary Table S1.

### 3.2. Serum sCD40 Levels Are Not Associated with Clinical Features in Patients with RA

**Figure 1:**
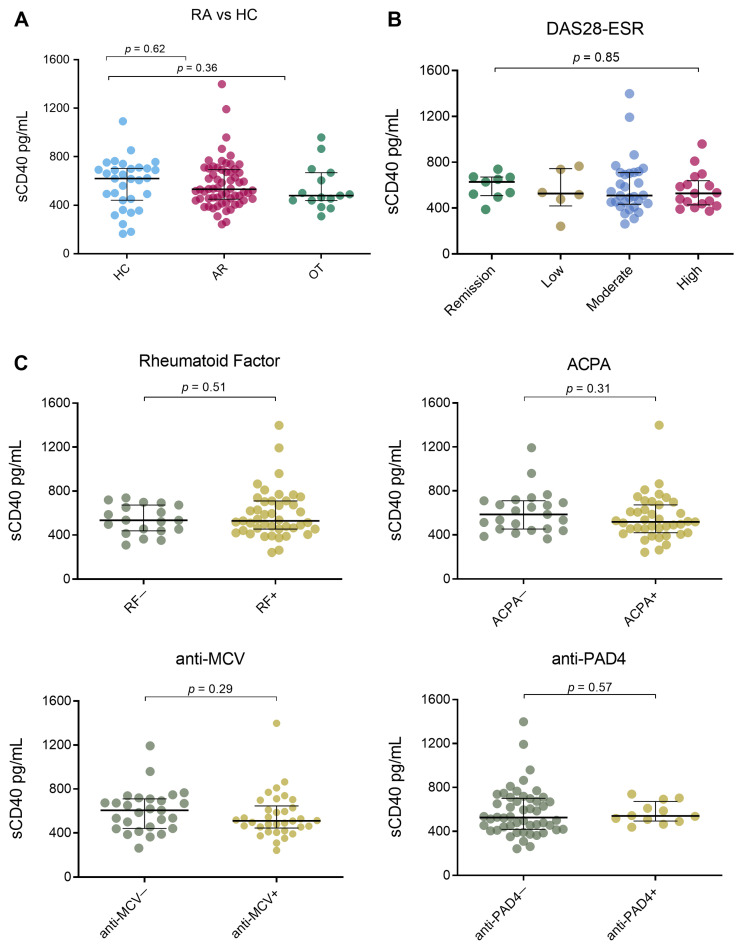
Serum sCD40 levels in patients with RA. (**A**) Serum sCD40 levels in healthy controls (HC, *n* = 31), patients with RA (*n* = 62), and off-treatment patients with RA (off-treatment: OT, *n* = 15). (**B**) Serum sCD40 levels in patients with RA stratified according to DAS28-ESR disease activity category (remission, *n* = 9; low disease activity, *n* = 7; moderate disease activity, *n* = 31; and high disease activity, *n* = 18). (**C**) Serum sCD40 levels according to seropositivity for rheumatoid factor (RF; negative, *n* = 19; positive, *n* = 43), anti-citrullinated peptide antibodies (ACPA; negative, *n* = 23; positive, *n* = 39), anti-mutated citrullinated vimentin antibodies (anti-MCV; negative, *n* = 27; positive, *n* = 34), and anti-peptidyl arginine deiminase 4 antibodies (anti-PAD4; negative, *n* = 50; positive, *n* = 12). Data are presented as the median (interquartile range). Comparisons between two groups were performed using the Mann–Whitney U test, whereas comparisons among disease activity categories were performed using the Kruskal–Wallis test.

Serum sCD40 levels were measured in patients with RA and healthy controls (HC). No significant differences were observed between the two groups [620.2 (440.8–702.8) vs. 531.4 (449.7–693.9) pg/mL; *p* = 0.62]. Similarly, sCD40 levels did not differ significantly between HC and the off-treatment (OT) subgroup [620.2 (440.8–702.8) vs. 479.3 (438.6–668.7) pg/mL; *p* = 0.36; Figure 1A]. When patients with RA were stratified by sex, no significant differences were observed between females and males [533.7 (453.0–674.2) vs. 488.5 (417.3–831.3) pg/mL; *p* = 0.79]. Likewise, sCD40 levels did not differ among patients classified according to DAS28-ESR disease activity categories [remission: 629.3 (509.2–670.8) pg/mL; low disease activity: 527.6 (419.3–744.4) pg/mL; moderate disease activity: 510.5 (433.6–709.8) pg/mL; high disease activity: 529.1 (429.2–639.8) pg/mL; *p* = 0.85; Figure 1B].

Spearman correlation analysis was performed to evaluate the association between sCD40 levels and clinical characteristics, including DAS28-ESR score, disease duration, and acute-phase reactants. No significant associations were identified. However, sCD40 levels showed a positive correlation with age (*r_s_* = 0.293, *p* = 0.02; Table 2). No significant correlation between age and sCD40 levels was observed in the HC group (*r* = 0.2595; *p* = 0.1586).

To determine whether treatment status influenced sCD40 levels, patients with RA were further stratified according to treatment. No significant differences were observed between OT patients [479.3 (438.6–668.7) pg/mL] and patients receiving active treatment [537.3 (455.5–702.9) pg/mL; *p* = 0.2812].

### 3.3. Serum sCD40 Levels Are Not Associated with Autoantibody Status in Patients with RA

Serum titers of RF, ACPA, anti-MCV, and anti-PAD4 autoantibodies were determined in patients with RA. When patients were stratified according to seropositivity, no significant differences in sCD40 levels were observed between RF-seropositive and RF-seronegative patients [RF+ 529.1 (453.7–709.4) vs. RF− 533.7 (438.6–673.0) pg/mL; *p* = 0.51]. Likewise, no significant differences were observed for ACPA [ACPA+ 518.0 (419.8–673.0) vs. ACPA− 586.1 (453.0–709.4) pg/mL; *p* = 0.31], anti-MCV [anti-MCV+ 510.5 (445.2–646.4) vs. anti-MCV− 605.4 (440.0–709.4) pg/mL; *p* = 0.29], or anti-PAD4 [anti-PAD4+ 540.1 (494.6–672.0) vs. anti-PAD4− 525.6 (418.4–700.7) pg/mL; *p* = 0.57; Figure 1C]. Spearman correlation analysis revealed a trend toward a negative correlation between sCD40 levels and anti-MCV titers (*r_s_* = −0.231, *p* = 0.07). No significant correlations were observed between sCD40 levels and RF, ACPA, or anti-PAD4 titers (Table 2).

Because seronegative patients with RA lack the characteristic autoantibody profile of the disease, sCD40 levels in this subgroup were also compared with those of HC. No significant differences in sCD40 levels were observed for any of the four autoantibodies evaluated: RF [533.7 (438.6–673.0) vs. 620.2 (440.8–702.8) pg/mL, *p* = 0.3719], ACPA [586.1 (453.0–709.4) vs. 620.2 (440.8–702.8) pg/mL, *p* = 0.8351], anti-MCV [605.4 (440.0–709.4) vs. 620.2 (440.8–702.8) pg/mL, *p* = 0.8893], and anti-PAD4 [525.6 (418.4–700.7) vs. 620.2 (440.8–702.8) pg/mL, *p* = 0.6602].

An additional analysis was performed considering patients’ treatment. The OT subgroup was stratified according to seropositivity for each autoantibody evaluated. No significant differences in sCD40 levels were observed for anti-MCV [471.8 (394.2–645.9) vs. 500.9 (438.6–668.7) pg/mL, *p* = 0.6943], ACPA [479.3 (414.0–651.6) vs. 470.5 (425.3–741.4) pg/mL, *p* = 0.9546], or anti-PAD4 [464.3 (438.6–490.1) vs. 490.1 (399.1–690.5) pg/mL, *p* = 0.6330]. This analysis could not be performed for RF because only one OT patient was seronegative (*n* = 1).

Similarly, among patients receiving active treatment, sCD40 levels did not differ significantly between seropositive and seronegative individuals for RF [537.3 (453.0–709.4) vs. 559.9 (475.0–666.0) pg/mL, *p* = 0.9853], anti-MCV [523.5 (446.8–647.7) vs. 619.6 (454.2–714.4) pg/mL, *p* = 0.3897], ACPA [525.6 (417.3–680.5) vs. 619.6 (484.1–714.4) pg/mL, *p* = 0.2479], or anti-PAD4 [587.0 (527.6–697.7) vs. 531.4 (418.4–709.8) pg/mL, *p* = 0.3296].

### 3.4. Serum sCD40 Levels Are Associated with Selected Cytokines in Patients with RA

Serum cytokine concentrations were quantified using a bead-based multiple assay in all study participants. Cytokine concentrations in the overall RA cohort, the OT subgroup, and healthy controls are summarized in Supplementary Table S2. Spearman correlation analysis was performed to evaluate the association between serum cytokine concentrations and sCD40 levels in patients with RA. As shown in Table 2, significant negative correlations were observed between sCD40 levels and IL-1β (*r_s_* = −0.307, *p* = 0.01), IL-2 (*r_s_* = −0.317, *p* = 0.01), IL-4 (*r_s_* = −0.355, *p* = 0.004), and TNF-α (*r_s_* = −0.285, *p* = 0.02).

To determine whether these associations were independent of age, four separate multiple linear regression models were fitted for IL-1β, IL-2, IL-4, and TNF-α, with age included as a covariate. Of the cytokines evaluated, only IL-2 remained significantly associated with serum sCD40 levels after adjustment for age [*B* = −1.698; 95% CI, −3.351 to −0.045; *p* = 0.044]. No significant associations were observed between sCD40 levels and IL-1β, IL-4, or TNF-α after age adjustment (Supplementary Table S3).

## 4. Discussion

Rheumatoid arthritis (RA) is characterized by chronic inflammation, autoantibody production, and elevated levels of proinflammatory cytokines [1,2]. Among the key molecular mechanisms underlying the initiation and progression of inflammation in RA are cell–cell interactions and the activation of costimulatory pathways. The CD40-CD40L signaling axis is particularly important because it contributes to the production of ACPA and proinflammatory cytokines—including TNF-α, IL-6, IL-17, IL-12, and IL-8—by key cell populations such as macrophages and synovial fibroblasts [3,4,5]. Additionally, CD40-mediated signaling in synovial fibroblasts promotes cell proliferation and increases the production of mediators involved in angiogenesis and bone resorption, including VEGF, matrix metalloproteinases (MMPs), and RANKL [3,4,5]. The soluble form of CD40 (sCD40) has been proposed to retain the ability to bind membrane-bound CD40L and act as a natural antagonist, or decoy receptor, of CD40-mediated signaling [6,33]. To date, the relationship between sCD40 and inflammatory or humoral markers in RA has not been investigated. Therefore, the present study aimed to evaluate serum sCD40 levels in patients with RA and examine their association with proinflammatory cytokines and autoantibody seropositivity.

Upon evaluating serum sCD40 levels in patients with RA, no significant differences were observed compared with healthy controls. This finding contrasts with our expectations, as elevated sCD40 levels have consistently been reported in several autoimmune diseases, including SLE, GD, HT, SSc, and psoriasis [17,18,19,34]. Furthermore, proteolytic cleavage and release of sCD40 by tumor necrosis factor-α-converting enzyme (TACE, also known as ADAM17) have been shown to be enhanced under inflammatory conditions [6,7]. Therefore, higher circulating sCD40 levels would be expected in patients with inflammatory diseases than in healthy individuals. Moreover, no significant differences in sCD40 levels were observed among patients stratified according to DAS28-ESR disease activity category. This finding further supports the notion that sCD40 release is not associated with the degree of inflammation in RA.

An interesting finding of our study was the positive correlation between serum sCD40 levels and age in patients with RA. To date, no studies have reported a similar association in the context of autoimmune inflammatory diseases. However, recent studies in cardiovascular disease have consistently demonstrated a positive association between circulating sCD40 levels and advancing age [7,35]. Notably, this age-related correlation was observed exclusively in RA patients, as no significant association between age and sCD40 levels was found in HC. This may be explained by prior findings in healthy adults showing that, while CD40L expression on T cells increases with age, CD40 expression on B cells decreases with age [36]. Since sCD40 is primarily generated through the shedding of membrane-bound CD40, the age-related decline in CD40 expression may reduce the amount of CD40 available for shedding, even though ADAM17 activity increases with age [37]. In RA, however, membrane CD40 expression remains chronically elevated due to ongoing inflammation, which may lead to a progressive increase in sCD40 levels as subjects age. It is important to note, however, that no studies have directly linked these two mechanisms to date.

In addition to proteolytic cleavage by TACE, sCD40 is also generated through alternative splicing of the CD40 gene [11,12]. The production of the soluble CD40 isoform is tightly regulated and is inversely related to the expression of its membrane-bound counterpart [11,12]. This regulatory process is influenced by key inflammatory stimuli, including IFN-γ and LPS, which modulate the balance between the soluble and membrane-bound CD40 isoforms [11,12]. Despite these insights, the molecular mechanisms regulating sCD40 production in RA remain poorly understood. Furthermore, the primary cellular source of sCD40 in RA has not yet been identified. This knowledge gap is particularly relevant because CD40 expression extends beyond immune cells and has also been reported in fibroblasts and endothelial cells. A better understanding of the cellular origin and regulation of sCD40 in RA may provide valuable insights into the mechanisms underlying this disease.

CD40 signaling has been shown to be essential for the production of IgM ACPA by B cells in both patients with RA and healthy individuals [38]. In contrast, the soluble form of CD40 has been identified as an inhibitor of immunoglobulin synthesis. This inhibitory effect results from the ability of sCD40 to compete with its membrane-bound CD40 for binding to CD40L, thereby reducing immunoglobulin (Ig) production by CD40L-activated B cells. Furthermore, sCD40 has been shown to selectively suppress CD40/CD40L-mediated Ig synthesis by B cells in vitro [6]. In addition to RF and ACPA, anti-MCV and anti-PAD4 autoantibodies have been proposed as markers of disease severity in RA [39,40]. Collectively, these autoantibodies reflect humoral immune activity in this disease. Therefore, we evaluated the relationship between sCD40 levels and seropositivity for RF, ACPA, anti-MCV, and anti-PAD4 autoantibodies. In contrast to findings reported in other autoimmune diseases, such as HT and GD [17], our results indicate that serum sCD40 levels are not associated with either the presence or titers of these autoantibodies in patients with RA. Notably, sCD40 levels in seronegative patients with RA did not differ from those observed in the HC group, further supporting the lack of association between sCD40 and autoantibody status in this disease.

This lack of association remained consistent after patients were further stratified according to treatment status (i.e., off-treatment or receiving active treatment), as neither treatment status nor autoantibody status within each treatment subgroup significantly influenced circulating sCD40 levels. Because all patients in our cohort received conventional synthetic DMARDs, whether these findings also apply to other treatment approaches, such as biologic DMARDs or Janus kinase (JAK) inhibitors, remains to be determined in larger and more heterogeneous cohorts.

The CD40/CD40L axis plays a central role in sustaining the inflammatory environment in RA by promoting the production of proinflammatory cytokines, B-cell differentiation and survival, and autoantibody production [41,42]. Regarding its expression throughout disease progression, both CD40 and CD40L have been reported to be upregulated in the synovial membrane of patients with early RA compared to healthy controls [43]. Moreover, we previously reported that CD40 expression on peripheral blood CD4+ T cells is higher in patients with early RA than in those with established disease [17]. Taken together, these findings suggest that the role of sCD40 in humoral immunity, and consequently in autoantibody production, may be more relevant during the early stages of the disease, when CD40 expression is increased and immunological tolerance is disrupted. However, further studies are needed to confirm this hypothesis.

CD40/CD40L signaling is a key mechanism driving the production of proinflammatory cytokines, including IL-6, IL-12, IL-17, IL-8, and TNF-α, by cell populations involved in the pathogenesis of RA, such as synovial fibroblasts, macrophages, B cells, and dendritic cells [3,4,5]. In the present study, we investigated the association between serum sCD40 levels and circulating cytokine concentrations in patients with RA. Significant negative correlations were observed between sCD40 and IL-1β, IL-2, IL-4, and TNF-α. These findings differ from those reported in T1DM, in which elevated sCD40 levels were detected in patients compared with healthy controls and were positively correlated with inflammatory markers such as CRP and IL-6 [44]. Although the direct effects of sCD40 on cytokine production remain poorly understood, its proposed role as a natural antagonist of the CD40/CD40L signaling axis suggests that the inverse associations observed in our study may reflect inhibition of this pathway. However, after adjustment for age using multiple linear regression models, only the association between sCD40 and IL-2 remained statistically significant. This finding suggests that the relationship between sCD40 and IL-2 is independent of age, whereas the association observed with IL-1β, IL-4, and TNF-α may have been influenced, at least in part, by age-related effects. Nevertheless, studies involving larger cohorts are needed to confirm these findings.

This study has several limitations that should be considered when interpreting the findings. First, its cross-sectional design precludes any inference of causal relationships between serum sCD40 levels and the inflammatory or humoral markers evaluated in patients with RA. Secondly, the relatively small sample size, particularly in the subgroup analyses, may have reduced the statistical power to detect significant associations and increased the risk of type II error. This was especially evident in the disease activity analyses, as most patients had moderate disease activity, whereas relatively few had high disease activity, potentially limiting the ability to detect differences associated with more severe disease. Similarly, stratification according to treatment status and subsequent autoantibody seropositivity resulted in only one RF-seronegative patient in the off-treatment subgroup, precluding this comparison. Third, the use of serum samples represents an important limitation, as circulating sCD40 levels may not accurately reflect CD40/CD40L activity within the synovial compartment. Consequently, the relationship between serum sCD40 levels and the local synovial inflammatory environment remains unclear. Fourth, treatment heterogeneity among patients receiving active therapy, all of whom were treated with conventional synthetic DMARDs in different therapeutic combinations, was not included as a potential confounding factor and should therefore be considered when interpreting our findings. Finally, although significant correlations between sCD40 and specific cytokines were identified, no functional assays were performed to investigate the biological relevance or mechanistic bases of these associations. Consequently, the mechanisms underlying these relationships remain to be elucidated.

Despite these limitations, this study provides a foundation for future research. The association between sCD40 and IL-2 after adjustment for age warrants further investigation in larger cohorts and prospective studies. Moreover, the absence of an association between sCD40 and RA-related autoantibodies does not necessarily exclude a role for the CD40/CD40L pathway in humoral immunity, as sCD40 may be more relevant during the early stages of the disease, before the autoantibody response is fully established. Longitudinal studies including individuals at risk of developing RA or patients with early disease would help address this question. Finally, functional studies are needed to clarify the biological significance and mechanistic basis of associations identified in the present study.

## 5. Conclusions

The findings of the present study indicate that sCD40 levels do not reflect the inflammatory or humoral status of RA, unlike what has been previously reported for the membrane-bound forms of CD40 and CD40L, as well as for soluble CD40L. Further studies are needed to clarify the functional role of sCD40 in this disease.

Exploring the potential modulatory functions of sCD40 within the inflammatory microenvironment may provide valuable insights into its mechanistic relevance. Specifically, future studies should determine whether sCD40 exerts regulatory or protective functions during distinct stages of RA and whether these effects are age-dependent. Addressing these questions will require comprehensive longitudinal and functional studies. Such investigations will help define the circumstances under which sCD40 may influence key molecular pathways involved in RA pathogenesis.

## Figures and Tables

**Table 1:** Demographic and clinical characteristics of patients with RA.

Variable	RA (*n* = 62)
Demographics	
Age (years) ^†^	46.44 ± 10.50
Sex (female/male) ^§^	51 (82.3)/11 (17.7)
Clinical characteristics	
Disease duration (years) ^†^	4.29 ± 5.98
CRP (mg/L) ^†^	61.42 ± 83.34
ESR (mm/h) ^†^	33.97 ± 18.52
DAS28-ESR ^†^	4.25 ± 1.43
Disease activity (DAS28-ESR) ^§^	
Remission (<2.6)	9 (14.52)
Low disease activity (≥2.6 < 3.2)	6 (9.68)
Moderate disease activity (≥3.2 < 5.1)	30 (48.39)
High disease activity (≥5.1)	17 (27.41)
RF (IU/mL) ^‡^	75.9 (28.45–243.3)
RF negative (<20 IU/mL) ^§^	19 (30.65)
RF positive (≥20 IU/mL) ^§^	43 (69.35)
ACPA (U/mL) ^‡^	22.6 (2.8–57.8)
ACPA negative (<5 U/mL) ^§^	23 (37.10)
ACPA positive (≥5 U/mL) ^§^	39 (62.90)
Anti-MCV (U/mL) ^‡^	49.6 (5.1–140.7)
Anti-MCV negative (<20 U/mL) ^§^	27 (43.55)
Anti-MCV positive (≥20 U/mL) ^§^	34 (54.84)
Anti-PAD4 (U/mL) ^‡^	2142 (1196–3681)
Anti-PAD4 negative (<4749 U/mL) ^§^	50 (80.65)
Anti-PAD4 positive (≥4749 U/mL) ^§^	12 (19.35)
Treatment ^§^	
NSAIDs	35 (56.45)
Corticosteroids ^¶^	23 (37.10)
DMARDs	
Methotrexate	41 (66.12)
Sulfasalazine	15 (24.19)
Chloroquine	6 (9.68)

^†^ Data are presented as mean ± SD; ^‡^ Data are presented as median (IQR); ^§^ Data are presented as *n* (%). Abbreviations: ACPA, anti-citrullinated peptide antibodies; Anti-MCV, anti-mutated citrullinated vimentin antibodies; Anti-PAD4, anti-peptidyl arginine deiminase 4 antibodies; CRP, C-reactive protein; DAS28, 28-joint Disease Activity Score; DMARDs, disease-modifying antirheumatic drugs; ESR, erythrocyte sedimentation rate; NSAIDs, nonsteroidal anti-inflammatory drugs; RF, rheumatoid factor. ^¶^ Corticosteroid doses were ≤15 mg/day in all patients.

**Table 2:** Correlation between serum sCD40 levels and clinical characteristics, cytokine concentrations, and autoantibody titers in patients with RA.

Variable	*r_s_*	95% CI	*p* Value
Age (years)	0.293	(0.037 to 0.513)	0.02
Clinical characteristics			
DAS28-ESR (score)	−0.172	(−0.410 to 0.088)	0.18
Disease duration (years)	0.041	(−0.220 to 0.296)	0.75
CRP (mg/dL)	−0.078	(−0.330 to 0.184)	0.54
ESR (mm/h)	−0.006	(−0.266 to 0.255)	0.96
Autoantibodies			
RF (IU/mL)	−0.063	(−0.314 to 0.197)	0.62
ACPA (U/mL)	−0.134	(−0.378 to 0.126)	0.29
Anti-MCV (U/mL)	−0.231	(−0.462 to 0.030)	0.07
Anti-PAD4 (U/mL)	0.089	(−0.171 to 0.338)	0.49
Cytokines (pg/mL)			
IL-1β	−0.307	(−0.522 to −0.0545)	**0.01**
IL-2	−0.317	(−0.531 to −0.065)	**0.01**
IL-4	−0.355	(−0.560 to −0.107)	**0.004**
IL-6	−0.109	(−0.355 to 0.152)	0.40
IL-10	−0.097	(−0.345 to 0.163)	0.45
IL-13	−0.199	(−0.433 to 0.061)	0.12
IL-12	−0.125	(−0.369 to 0.136)	0.33
IL-17	0.0013	(−0.255 to 0.258)	0.99
IFN-γ	0.1030	(−0.158 to 0.350)	0.42
TNF-α	−0.285	(−0.505 to −0.030)	**0.02**

Abbreviations: ACPA, anti-citrullinated peptide antibodies; Anti-MCV, anti-mutated citrullinated vimentin antibodies; Anti-PAD4, anti-peptidyl arginine deiminase 4 antibodies; CI, confidence interval; CRP, C-reactive protein; DAS28, 28-joint Disease Activity Score; ESR, erythrocyte sedimentation rate; RF, rheumatoid factor. Spearman’s rank correlation coefficient (*r_s_*) was used for all correlation analyses. Bold values indicate statistically significant differences (*p* < 0.05).

## Data Availability

The original contributions presented in this study are included in the article. Further inquiries may be directed to the corresponding author.

## Supplementary Materials

The following supporting information can be downloaded at: https://www.mdpi.com/article/10.3390/jcm15155836/s1, Table S1: Demographic and clinical characteristics of OT patients with RA and healthy controls; Table S2: Serum cytokine levels in the overall RA cohort, OT patients with RA, and healthy controls; Table S3: Results of independent multiple linear regression models evaluating the association between serum sCD40 levels and cytokines.

## Abbreviations

The following abbreviations are used in this manuscript:

ACPA	Anti-citrullinated peptide antibodies
ACR	American College of Rheumatology
ADAM	A disintegrin and metalloproteinase
Anti-MCV	Anti-mutated citrullinated vimentin antibodies
Anti-PAD4	Anti-peptidylarginine deiminase 4 antibodies
ASST	Autologous serum skin test
aTSHR	Anti-thyroid-stimulating hormone receptor antibodies
CD40	Cluster of differentiation 40
CD40L	Cluster of differentiation 40 ligand
CRP	C-reactive protein
HC	Healthy controls
CU	Chronic urticaria
DAS28-ESR	28-joint Disease Activity Score based on erythrocyte sedimentation rate
DMARDs	Disease-modifying antirheumatic drugs
ELISA	Enzyme-linked immunosorbent assay
ESR	Erythrocyte sedimentation rate
EULAR	European Alliance of Associations for Rheumatology
GD	Graves’ disease
HT	Hashimoto’s thyroiditis
IFN-γ	Interferon-γ
Ig	Immunoglobulin
IL	Interleukin
LPS	Lipopolysaccharide
MCP-1	Monocyte chemoattractant protein 1
MIP-1α	Macrophage inflammatory protein 1 alpha
MMP	Matrix metalloproteinase
mRNA	Messenger ribonucleic acid
NSAIDs	Non-steroidal anti-inflammatory drugs
OT RA	Off-treatment patients with rheumatoid arthritis
RA	Rheumatoid arthritis
RANKL	Receptor activator of nuclear factor kappa-B ligand
RF	Rheumatoid factor
sCD40	Soluble CD40
SLE	Systemic lupus erythematosus
SSc	Systemic sclerosis
T1DM	Type 1 diabetes mellitus
TACE	Tumor necrosis factor-alpha converting enzyme
TNF-α	Tumor necrosis factor-alpha
TNFSF	Tumor necrosis factor superfamily
TNFRSF	Tumor necrosis factor receptor superfamily
VEGF	Vascular endothelial growth factor
