# Supplementary material for: Soluble CD40 Levels in Rheumatoid Arthritis: Association with Serum Cytokines and Autoantibody Status
Source: J Clin Med. 2026 Jul 26;15(15):5836. doi: 10.3390/jcm15155836 (PMC13466420; doi:10.3390/jcm15155836)
Supplement: Supplementary file 1 [file jcm-15-05836-s001.zip › jcm-4423157-supplementary.pdf]

Supplementary Table S1. Demographic and clinical characteristics of OT patients with RA and healthy controls.

Variable	OT RA (*n* = 15)	HC (*n* = 31)
**Demographics**		
Age (years)†	41.50 ± 10.52	42.65 ± 9.97
Sex (female/male)‡	14 (93.3) / 1 (6.7)	27 (87.1) / 4 (12.9)
**Clinical characteristics**		
Disease duration (years)†	2.29 ± 2.88	-
CRP (mg/L)†	77.75 ± 98.47	2.33 ± 3.55
ESR (mm/h)†	29.53 ± 15.53	21.14 ± 11.24
DAS28-ESR†	4.96 ± 1.08	-
Disease activity (DAS28-ESR)‡		-
Remission (< 2.6)	1 (6.7)	-
Low disease activity (≥ 2.6 < 3.2)	0 (0.0)	-
Moderate disease activity (≥ 3.2 < 5.1)	8 (53.3)	-
High disease activity (≥ 5.1)	6 (40.0)	-
**RF (IU/mL)†**	**125.59 ± 119.43**	-
RF negative (< 20 IU/mL)‡	1 (6.7)	-
RF positive (≥ 20 IU/mL)‡	14 (93.3)	-
**ACPA (U/mL)†**	**36.44 ± 53.77**	-
ACPA negative (< 5 U/mL)‡	6 (40.0)	-
ACPA positive (≥ 5 U/mL)‡	9 (60.0)	-
**Anti-MCV (U/mL)†**	**197.7 ± 356.56**	-
Anti-MCV negative (< 20 U/mL)‡	7 (46.7)	-
Anti-MCV positive (≥ 20 U/mL)‡	8 (53.3)	-
**Anti-PAD4 (U/mL)†**	**2564.99 ± 2058.64**	-
Anti-PAD4 negative (< 4749 U/mL)‡	1 (6.7)	-
Anti-PAD4 positive (≥ 4749 U/mL)‡	14 (93.3)	-

†Data are presented as mean ± SD; ‡Data are presented as *n* (%). Abbreviations: ACPA, anti-citrullinated peptide antibodies; Anti-MCV, anti-mutated citrullinated vimentin antibodies; Anti-PAD4, anti-peptidyl arginine deiminase 4 antibodies; CRP, C-reactive protein; HC, healthy controls; DAS28, 28-joint Disease Activity Score 28; ESR, erythrocyte sedimentation rate; OT RA, off-treatment rheumatoid arthritis patients; RF: rheumatoid factor.

Supplementary Table S2. Serum cytokine levels in the overall RA cohort, OT patients with RA, and healthy controls.

	**Total RA (n=62)**	**OT RA (n=15)**	**HC (n=31)**	***p* value‡**	***p* value§**
**Cytokines (pg/mL) †**					
IL-1β	23.78 (10.0-131.0)	60.65 (10.05-166.50)	12.50 (10.00-25.92)	0.071	**0.009**
IL-2	3.75 (0.33-16.40)	5.51 (3.31-34.15)	0.33 (0.33-5.76)	0.118	**0.01**
IL-4	12.92 (10.0-49.32)	28.95 (10.00-137.30)	10.0 (10.0-20.45)	0.112	**0.002**
IL-6	7.02 (0.80-28.34)	19.58 (5.03-34.86)	0.80 (0.80-6.09)	**0.011**	**0.0003**
IL-10	16.08 (13.14-20.72)	14.08 (11.48-35.31)	14.08 (13.05-20.12)	0.237	0.84
IL-13	12.0 (12.0-58.33)	65.60 (12.0-101.50)	12.0 (12.00-37.09)	0.637	**0.04**
IL-12	192.4 (117.6-313.90)	219.80 (160.10-412.80)	151.50 (110.80-196.70)	0.075	**0.01**
IL-17	6.82 (6.82-10.83)	10.50 (6.07-13.34)	6.82 (3.73-8.08)	0.153	0.05
IFN-γ	3.0 (1.80-3.00)	3.0 (1.27-3.00)	3.0 (1.80-3.00)	0.289	0.21
TNF-α	4.40 (1.00-23.16)	22.22 (4.67-38.36)	1.0 (1.0-6.83)	0.156	**0.003**

†Data are presented as median (interquartile range). Abbreviations: HS, healthy controls; OT RA, off-treatment rheumatoid arthritis patients. Comparisons between groups were performed using the Mann-Whitney U test.

‡Overall RA cohort vs HC

§OT RA vs HC

Supplementary Table S3. Results of independent multiple linear regression models evaluating the association between serum sCD40 levels and cytokines.

Model	Predictor	B (unstandardized)	SE	95% CI	*p*-value
1	IL-1β	-0.299	0.160	(-0.619) — (0.020)	0.066
2	IL-2	-1.698	0.826	(-3.351) — (-0.045)	**0.044**
3	IL-4	-0.224	0.165	(-0.554) — (0.106)	0.179
4	TNF-α	0.031	0.279	(-0.528) — (0.591)	0.912

Abbreviations: *B*, unstandardized regression coefficient; SE: standard error; CI: confidence interval.
Each model was fitted independently, with serum sCD40 level as the dependent variable and the indicated cytokine together with age as independent variables. Statistically significant results ($p < 0.05$) are shown in bold.
